# Case report: Vascular graft infection due to *Aspergillus* species presenting with recurrent vascular occlusion

**DOI:** 10.1186/s12872-022-02571-3

**Published:** 2022-04-01

**Authors:** Ayami Tano, Koichi Kato, Yoshimasa Seike, Hitoshi Matsuda, Takashi Suzue, Yoshihiro Kaneko, Misato Kodama, Yuichi Sawayama, Akashi Miyamoto, Noriaki Yagi, Yoshihisa Nakagawa

**Affiliations:** 1 Department of Cardiovascular Medicine, Shiga University of Medical Science, Otsu, Japan; 2 Department of Cardiovascular Surgery, National Cerebral and Cardiovascular Center, Suita, Japan

**Keywords:** Graft infection, *Aspergillus*, Embolism, [18F]fluorodeoxyglucose positron emission tomography/computed tomography, Non-cultural study, β-D glucan, *Aspergillus* galactomannan antigen test

## Abstract

**Background:**

An aortic graft implantation is an effective therapeutic method for various aortic diseases. However, it is known that sometimes these implanted grafts can be the foci of infections. Here we report a rare case of graft infection that presented multiple embolisms of aortic branches and peripheral organs.

**Case presentation:**

A 63-year-old Japanese woman with a history of aortic graft implantation presented with occlusions of large arteries in different loci and time points, with elevation of non-specific inflammatory markers. Thoracic contrast-computed tomography (CT) captured vegetation in the descending aortic graft and the [18F]fluorodeoxyglucose positron emission tomography/computed tomography ([18F]FDG PET/CT) showed accumulation of FDG in the same site, suggesting a graft infection. Despite all these suspicious findings, repeated blood culture examinations never detected any microorganisms. A diagnosis of *Aspergillus* graft infection was made based on an elevated serum β-D glucan (βDG) and a positive *Aspergillus* galactomannan (GM) antigen test. The patient subsequently had surgery with replacement of the descending aortic graft and anti-fungal drugs were instituted with significant improvement noted.

**Conclusion:**

In the present case, the patient’s specific feature in the anatomical vascular construction, past operation, and basal fundamental diseases collaboratively contributed to the pathogenesis of the present infection. It is important to recognize the risk of graft infection and conduct imaging studies when indicative symptoms emerge. The negativity in blood culture studies often makes detection of pathogenic microbes extremely difficult. This case suggests that non-cultural tests such as bDG and GM can be useful for diagnosis and starting appropriate anti-fungal drugs in the early stages.

## Background

Artificial aortic replacement or thoracic abdominal endovascular aortic repair (TEVAR/EVAR) has been widely adopted as a treatment to reconstruct the aorta in various diseases. However, post-operative end-graft infection has been one of the major adverse events during management [1], which occurs in 0.4–5.0% of implanted cases [2–4]. Because of the high mortality rate reaching to 8–36% [1], establishing a consensus in diagnosis and therapeutic strategy is an urgent issue. Common clinical presentations of graft infections are abdominal or back pain, fever, leukocytosis, and sepsis. Some of them are derived from vascular sequelae such as plasticity and rupture of aneurysm, or aortic fistula to the trachea and gastrointestinal tracts [5–7]. *Staphylococci* and methicillin-sensitive *Staphylococcus aureus* have been reported as major pathogens, while fungal induced infection is known to be rare [5]. Diagnoses of graft infections are difficult, especially when unusual complications or pathogens exist. Here we report a unique case of an end-graft *Aspergillus* infection that presented repetitive embolization of large arteries, demonstrating the difficulty in diagnosing such cases.

## Case presentation

**Fig. 1:**
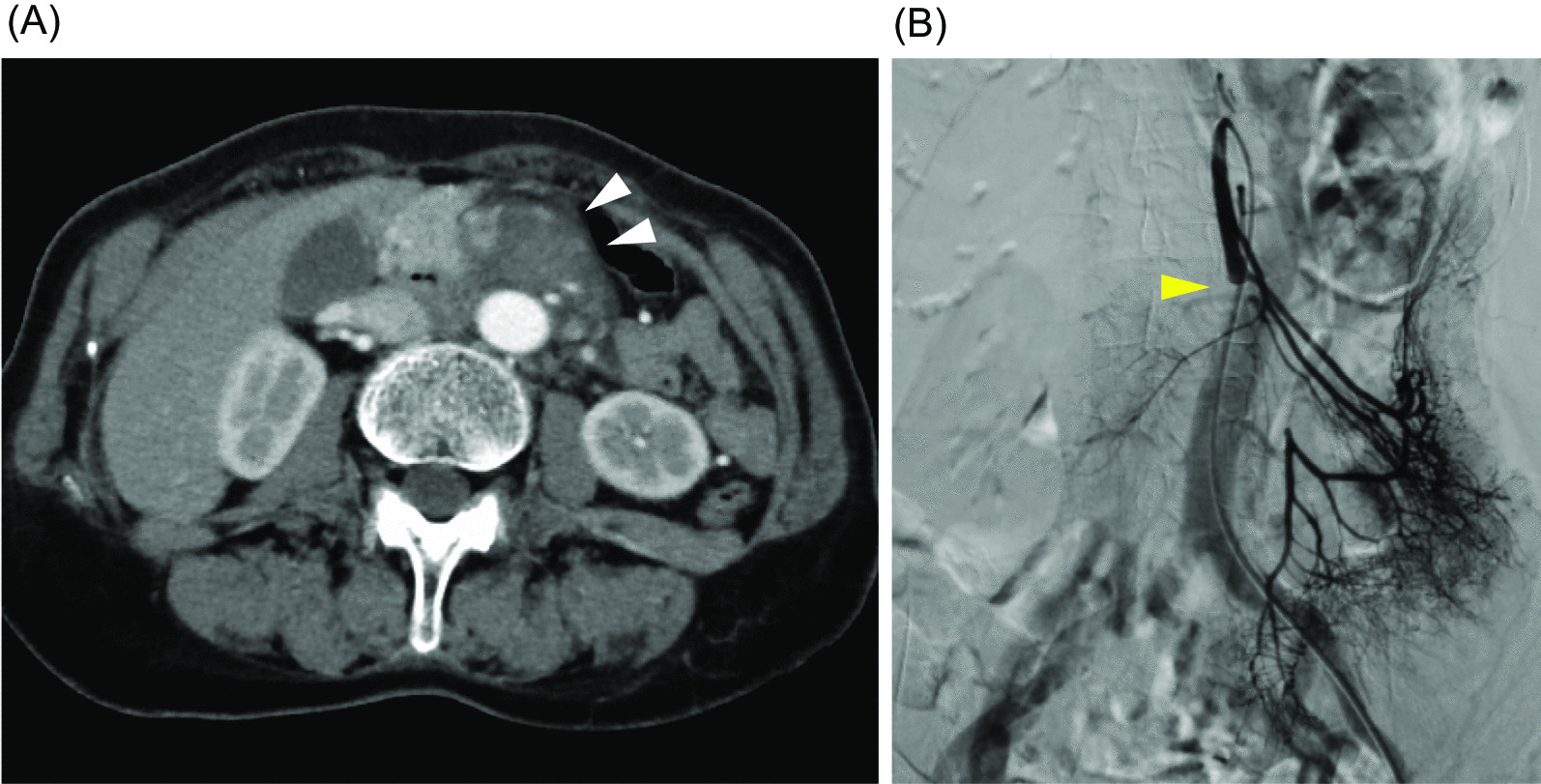
Imaging studies performed at first admission. **A** Completely obstructed SMA and swollen mesentery (white arrows) in a CT scan. **B** Abdominal angiography presented total occlusion at the first branch of the SMA (yellow arrow)

A 63-year-old woman known with aortic replacement/graft for coarctation of the aorta done 30-year ago and mitral valve replacement for mitral valve replacement done 6-month ago was admitted to our hospital. She presented with a 2-day history of nausea and persistent abdominal pain. On physical examination abdominal tenderness and impaired bowel sounds were noted. In blood tests, C-reactive protein (CRP) and D-dimer increased above the upper limit (12.78 mg/dL [0.00–0.03 mg/dL] and 1.5 μg/mL [0.0–0.9 mg/mL], respectively). The rest of the laboratory tests which including complete blood counts and chemistry panels done on admission were unremarkable. An abdominal contrast-enhanced CT showed embolism in the stem of first jejunum branch of the superior mesenteric artery (SMA) with disruption of the distal blood flow, and inflammatory changes in soft tissues around the SMA (Fig. 1A). Contrast enhancement of the bowel wall was normal. An abdominal angiography showed that nutrient branches from the SMA to the ileocecal and ascending colons were completely disrupted, and the collateral blood flow from the inferior mesenteric artery maintained blood supply to the intestinal walls (Fig. 1b). Though we screened the possible causes of arteritis or thrombosis, the tests including IgG4, antiphospholipid antibodies, anti-neutrophil cytoplasmic antibodies, antinuclear antibodies, treponemal antibody, and serological test for syphilis were all negative. We observed her on drip infusion for bowel resting and acetaminophen for pain control. On the 7th day, her abdominal symptoms were relieved. She was afebrile during the hospitalization. She could eat meals without any problems and was discharged on the 10th day.

**Fig. 2:**
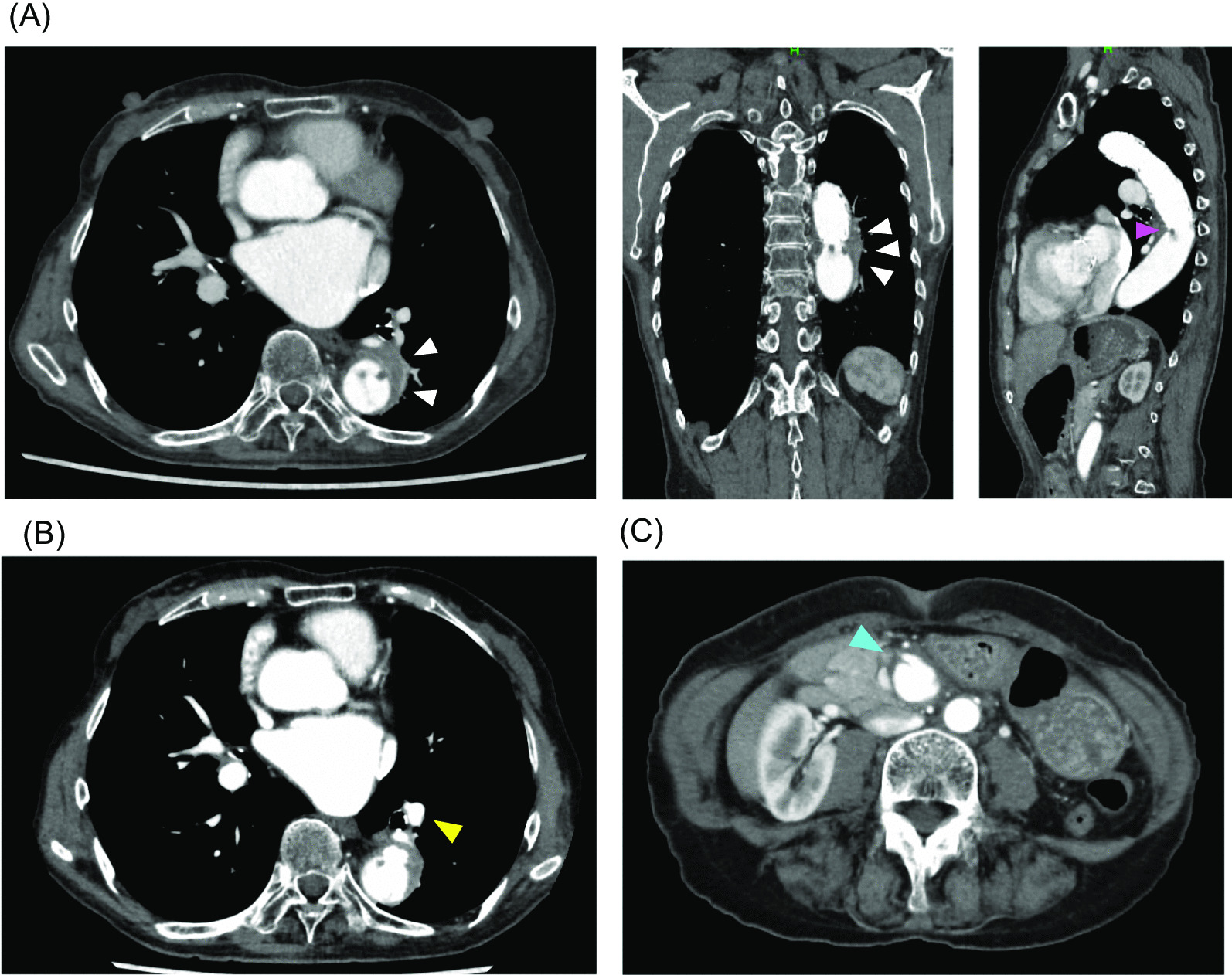
The thoracic and abdominal enhanced-CT in the second admission due to hemoptysis. **A** A pseudo aneurysm surrounding the distal end of the descending aortic graft (white arrows). The areas without CT-enhancement in the graft lumen indicate thrombus formation. A magenta arrow indicates the bent junction of the descending graft and aorta. **B** Formation of the fistula from the pseudo aneurysm to pulmonary vein and trachea (yellow arrow). **C** Enlargement of pseudo aneurysm at the obstructed part of the SMA (cyan arrow)

She was re-admitted a month later now presenting with hemoptysis. The blood tests again showed elevated CRP and D-dimer above the upper limit (3.26 mg/dL [0.00–0.03 mg/dL] and 2.8 μg/mL [0.0–0.9 mg/dL], respectively). A contrast-enhanced CT showed a cylindrical-shaped area lacking contrast enhancement inside the descending aortic graft and enhanced pseudo aneurysm around the distal end of the graft (Fig. 2A). That pseudo aneurysm formed fistula to pulmonary vein and trachea (Fig. 2B) which was considered for the cause of the hemoptysis. We also found an enlarged pseudo aneurysm at the part of the SMA that was obstructed one month before (Fig. 2C). These findings were strongly suggestive of the graft infection and vegetation growth. Since the pseudo aneurysm in the SMA was thought to be associated with infectious embolisms from the graft in the descending aorta, we started intravenous infusion of empiric antibiotics with ampicillin/sulbactam and daptomycin. Two sets of blood culture before the introduction of antibiotics were negative. On the day of admission and on the 4th day, we conducted coil embolization therapies to the SMA aneurysm twice to prevent enlargement and rupture. For the pseudo aneurysm in the descending aorta, we performed an additional zone 4 TEVAR. A transesophageal echocardiography during the operation showed a moving flap along the graft. During perioperative care, serum β-D glucan (βDG) values, which we examined because empiric anti-bacterial agents were ineffective, were found to be approximately 80 pg/mL [< 20 mg/dL]. Unexpectedly, two sets of blood culture specimens collected from the peripheral veins and intra aneurysm did not develop any pathogens. However, based on the high bDG values, we empirically administered micafungin, then switched to voriconazole due to an allergic skin rash. βDG values decreased to approximately 20 pg/mL after the anti-fungal therapy, and she was discharged. She was then followed up in outpatient with an oral anti-fungal drug: 100 mg per day of fluconazole (FLCZ).

**Fig. 3:**
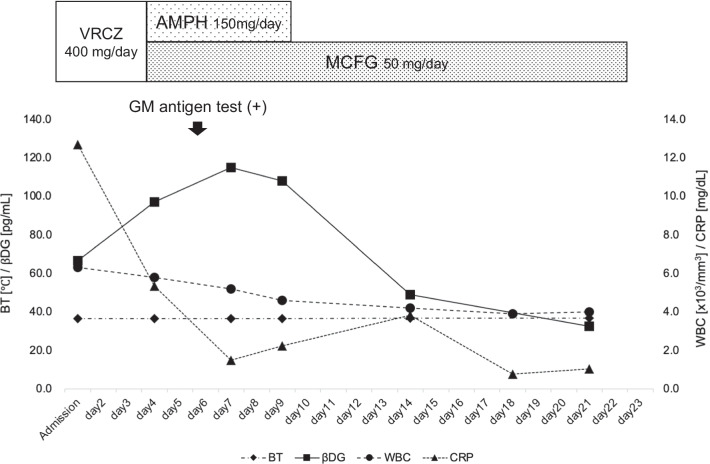
The therapeutic process during anti-fungal therapy in the 3rd admission. VRCZ; voriconazole; AMPH: amphotericin B; MCFG: micafungin; GM; galactomannan; BT: body temperature; βDG: β-D glucan [< 20 mg/dL]; WBC: white blood cells [3.0–8.0 × 10^3^/mm^3^]; CRP: C reactive protein [0.00–0.03 mg/dL]

**Fig. 4:**
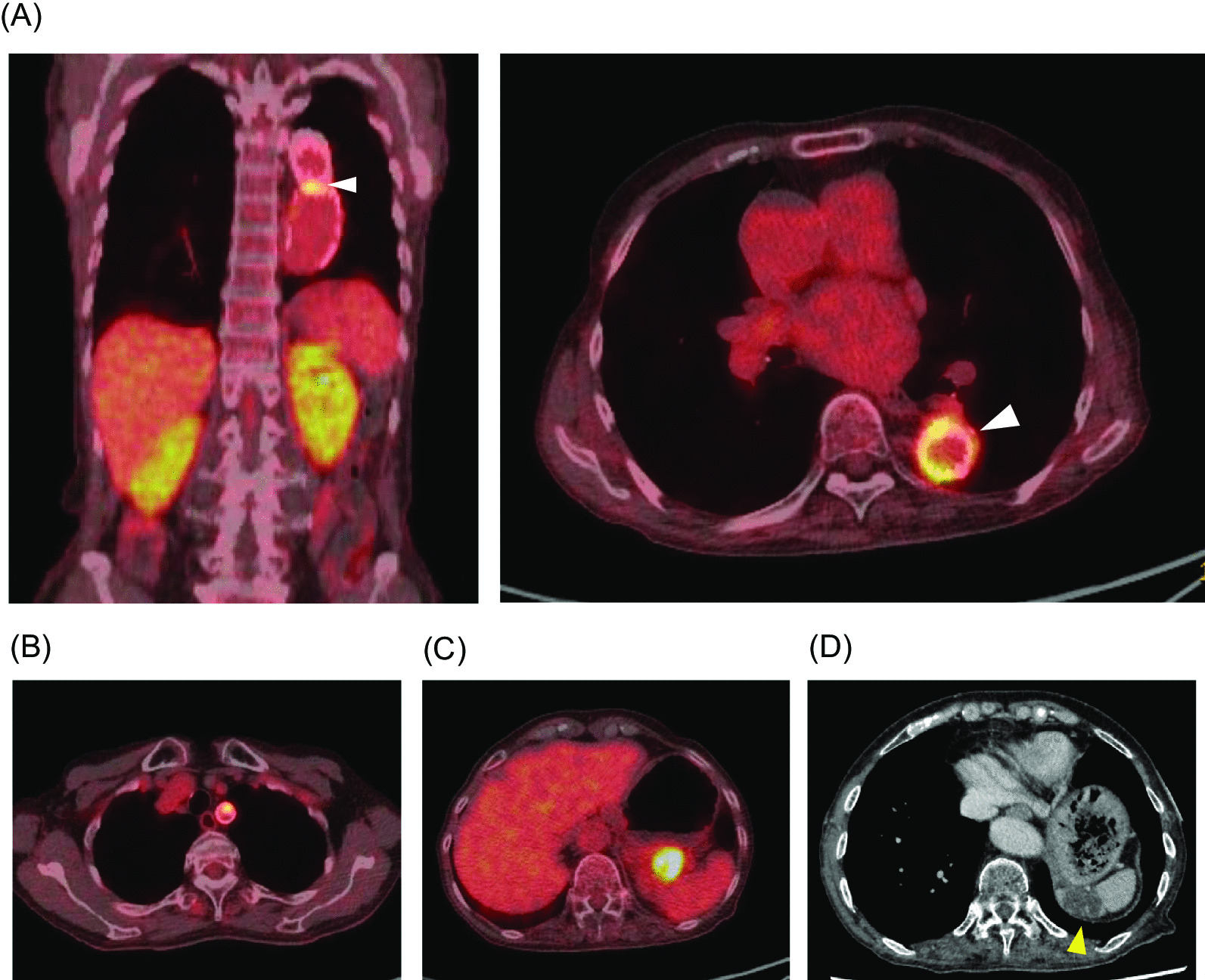
[18F]fluorodeoxyglucose positron emission tomography / computed tomography conducted to detect infectious foci. Accumulation of FDG is indicated: **A** in the distal end of the descending aortic graft in the coronal (left) and axial (right) sections (white arrows); **B** in the subclavian arterial root in the axial section; and **C** thromboembolism in the splenic artery. **D** Splenic infarctions detected by a CT scan (yellow arrow)

Seven and half months after the last admission, her abdominal pain recurred. An enhanced-CT on admission showed a newly developed aneurysm in the splenic artery and partial defects of staining suggesting splenic ischemia. She had been continued an oral anti-fungal drug since the last admission. We switched from an oral anti-fungal drug to intravenous infusion. We started the infusion with 400 mg per day of voriconazole (VRCZ) for the first four days, but had to change to 50 mg per day of micafungin (MCFG) and 150 mg per day of amphotericin B (AMPH) because of drug-induced hyponatremia. The patient’s condition improved and her inflammatory marker decreased over the course of the anti-fungal therapy (Fig. 3). For the splenic arterial aneurysm, we conducted a coil embolization. The repeated blood culture tests did not detect any pathogens, whereas the repeated βDG value increased up to 115.1 pg/mL and *Aspergillus* galactomannan (GM) antigen test was positive. Thus, we thought that *Aspergillus* species were the most suspicious pathogens. Additional [18F]fluorodeoxyglucose positron emission tomography / computed tomography ([18F]FDG PET/CT) showed accumulation of FDG in the distal end of the descending aortic graft (Fig. 4A), the root of subclavian artery (Fig. 4B), and the main trunk of a spleen artery (Fig. 4C). These accumulations indicated probable infections. Furthermore, a CT scan confirmed splenic infarctions (Fig. 4D). To remove these infected lesions, her grafts in the aortic arch and total descending aorta were surgically replaced. After establishment of an artificial heart–lung system via median sternotomy, we added a fifth-intercostal incision. We isolated the infected graft from the surrounding tissues carefully due to severe adhesion, then the graft was totally removed and a new graft was inserted (Ante-Flo 1Branch 22 mm®, Terumo, Japan). We could not detect any microbe in culture study of extracted graft. Six months after discharge, she is doing well under continuous oral anti-fungal therapy.

## Discussion and conclusion

Graft infections caused by fungus are quite rare, and the incidence reported in the literature is less than 10% of all graft infections [5, 8, 9]. The number of reported cases of graft infections by *Aspergillus* species was only 15 or less between 1980 and 2001 [10, 11].

Detection of pathogenic microorganisms is a gold standard for correct diagnoses and appropriate therapies for graft infections. However, specifically, *Aspergillus* species are known to be poorly detectable in blood culture studies [10–13]. Instead, a fungal antigen marker βDG can detect invasive Aspergillosis with sensitivity of 76%. When combined with *Aspergillus* GM antigen test, specificity of the test increases to 98% [14], which can be useful in making clinical decisions. In the present case, we used these tests to determine the pathogenic microbes. The positive *Aspergillus* GM antigen test as well as elevation of βDG values along with clinical symptoms provided evidence for *Aspergillus* infection, which enabled us to select anti-fungal drug with appropriate spectrum even though the multiple culture studies were negative.

To detect the infectious loci, we first narrowed the target to the descending aorta depending on the distribution of repetitive embolisms. The patient experienced recurrent embolisms in the areas fed by the SMA and splenic artery but never in cerebral arteries. Therefore, we excluded the involvement of the ascending aortic graft as an origin of emboli. Infectious endocarditis (IE) was also excluded by the same reason though this patient had a history of MVR. The CT and [18F]FDG PET/CT played important roles in detecting the infectious loci. [18F]FDG PET/CT is known to be useful to detect foci of graft infections with a sensitivity of 85% and a specificity of 68.4% [15]. Unlike our patient, most cases with graft infection had their infectious foci in the ascending aortic or arch grafts, which was probably because of more turbulent flow in these areas. We considered the atypical infectious foci in our case were because of the unique structural features of the patient’s aorta. In particular, the descending aortic graft was connected to the patient’s own aorta, forming a nested structure, and their junction was bent for the graft’s elasticity, as observed in the CT image (Fig. 2A, right). A turbulent blood flow caused by these complex structures may be an explanation for fungal growth at the site.

This case is also notable for remote onset of infection after an aortic graft insertion. In many reports, end-graft infections occurred during the early phase after insertion of the graft [16]. However, a year before the first admission, the patient had received MVR. Thus, there is a possibility that the open chest surgery caused the graft infection. MVR has been reported to cause IE, and cumulative IE risk after MVR within 10 years is reported to be 5.2% [17].

Current therapeutic options for graft infections are surgical and medication therapies [18]. Surgical interventions may be more curable than medication therapies, but they are excessively invasive. Most vascular grafts were not designed to be exchanged after the first placement, and one study showed that the mortality rate was 18–30% when grafts were surgically replaced [18]. Regarding graft infections after TEVAR/EVAR, another study in 2011 reported there was no significant difference in mortality rate between surgical and medication therapies [19]. On the other hand, if surgical replacement is not applicable, patients need to receive long-term broad-spectrum antibiotic therapy, which could cause drug-induced complications [20]. For those reasons, interventional strategies should be critically determined depending on conditions and underlying diseases of each patient. In our case, we thought a surgical approach was the most appropriate way because: (1) the patient was still tolerant for surgical replacement of the infected graft; and (2) oral anti-fungal medication could not prevent the recurrence of arterial embolism.

In conclusion, we experienced a rare case of repeated large-arterial embolisms originating from late-onset end-graft infection in the descending aorta. For the management of complex graft infection as in the present case, this report highlights (1) the importance of repeated imaging studies by using all available modalities including [18F]FDG PET/CT to specify the infectious loci, and (2) the usefulness of non-cultural tests for diagnosis and selection of effective agents, especially in case of fungal infection.

## Data Availability

The datasets used and/or analysed during the current study are available from the corresponding author on reasonable request.

## Abbreviations

βDG: β-D glucan
GM: Galactomannan
CRP: C-reactive protein
CT: Computed tomography
[18F]FDG PET/CT: [18F]fluorodeoxyglucose positron emission tomography/computed tomography
TEVAR: Thoracic endovascular aortic repair
EVAR: Endovascular aortic repair
SMA: Superior mesenteric artery
IE: Infective endocarditis
MVR: Mitral valve replacement
VRCZ: Voriconazole
AMPH: Amphotericin
MCFG: Micafungin
FLCZ: Fluconazole
